# On Neuralgia; with the Report of Two Cases of Excision of the Nerve

**Published:** 1856-12

**Authors:** John A. Lidell

**Affiliations:** Demonstrator of Anatomy to College of Physicians and Surgeons, New York; Surgeon to Bellevue Hospital, etc.


### *On Neuralgia; with the Report of two Cases of Excision of the Nerve.*

By John A. Lidell, M. D., Demonstrator of Anatomy to College of Physicians and Surgeons, New York; Surgeon to Bellevue Hospital, etc.

Operations for the relief of neuralgia are perhaps not sufficiently esteemed by surgeons at the present day. "Experience," says Miller, "has proved, that the relief, if any, obtained by such operations, is but partial and temporary, and the neuromatous enlargements, which form on the truncated extremities of the nerve, are likely to produce ultimate aggravation. The operation, in truth, may be the means of converting an example of neuralgia, unconnected with structural change in any part of the nerve, into a worse form, dependent on structural change, not only considerable, but probably irremediable. Sometimes the operation has proved successful upon the nerve, only to drive the neuralgia to another —perhaps inaccessible." Grant that such untoward results have followed both the simple division and the removal of a portion of the affected nerve. But do they *always* succeed these operations? Do they stand to each other *constantly* in the relation of cause and effect? Most certainly they do not, for it has happened many times that agonizing sufferers have derived both complete and permanent relief from their pains by the aid of a surgeon's knife, after all other means had entirely failed. In these cases, no one can doubt that operative interference is not only justifiable, but demanded by humane considerations. The simple division and even the removal of portions of nerves affected by neuralgia are, therefore, operations which should not be ignored by the surgeon, whose duty it is to treat the more aggravated and unmanageable forms of this distressing complaint.

Since experience has proved that the surgeon's knife cannot relieve all cases of neuralgia, the question naturally arises, to what class of cases the knife is applicable, or, in other words, what are the indications for operative interference. This question has been fitly answered by Mr. Erichsen, for he says that an operation "can only be of service when the pain is peripheral, occasioned by some local irritation existing between the part cut and the terminal branches of the nerve."

Apropos to this statement is the case related by Mr. Abernethy, of a lady who suffered from a neuralgia affecting the parts about the inner edge of the nail of the ring finger of the left hand. In course of time, the pain "extended all up the nerves of the arm," and, after eleven years of suffering, a cure was effected by cutting down upon, and removing a portion of, "the nerve of the finger from which all this disorder seemed to originate."

Mr. Lawrence, also, in a case of neuralgia resulting from a wound of the finger, excised a portion of the nerve "with permanent success." And Sir Astley Cooper completely cured a patient having "severe pain in the thumb, extending up the arm to the neck, and causing a distortion of the neck, fits," etc., by cutting down upon the radial nerve by the side of the flexor carpi radialis longus, and excising about five-eighths of an inch of it.

It will be observed that, in all these cases, the nerve was excised between the seat of irritation and the nervous centre; that the object of the operation was to break the line of communication between the painful part and the sentient being. This lesson from experience is in perfect accordance with the teachings of physiology, and furnishes, I apprehend, an important fact for the guidance of the operative surgeon. If the nerve can be excised between the seat of irritation and the nervous centre, so that an impression cannot be conveyed from the affected part to the sensorium, then will an operation be attended with success, and a cheering prospect may be held out to the suffering patient.

But, unfortunately, an operation cannot always be performed under these favorable conditions. Unfortunately the local mischief productive of the neuralgia may exist in the brain itself; in the track of the nerve, before its exit from the cranium; in the foramen through which it escapes, and even in remote parts of the body. In all such cases the knife cannot afford any rational prospect of cure, and, in the lighter forms of the disease, may even be productive of mischief. A curious fact may, however, be mentioned in this connection. It sometimes happens in cases where the nerve is not accessible between the seat of irritation and the nervous centre, that excision performed exterior to the seat of irritation affords relief, extending through weeks and even months. Might not the operation of excision be applicable to an extreme case of tic douloureux of this character, and of long standing, all other means of relief having failed? Might it not be justifiable and even humane to afford the patient this chance of temporary respite from suffering?

I have been led to make these reflections by a very severe case of tic douloureaux, which first came under my notice about the 20th of September, 1854. The patient, a woman, was in the medical division of Bellevue Hospital, under charge of Dr. George T. Elliot, one of the physicians to that charity, who kindly invited me to see her in consultation. She appeared to be about the middle period of life, pale, and emaciated. Her countenance was distorted with the severest suffering which ever came under my observation. Her mouth was drawn somewhat to the right side, and the muscles of that side of the face were twitching spasmodically. She was rubbing her cheek, just anterior to the ear, very diligently with cold water for the purpose of obtaining relief. In a few minutes the paroxysms passed away, but she had scarcely commenced to reply to my questions wheu it returned with great intensity. After a little while she succeeded in informing me that the pain, intense, "almost driving her crazy," and cutting in its character, always commenced in the right side of the lower lip, a little above the mental foramen, shot along the canal of the lower jaw to the front of the ear, and radiated somewhat over the right side of her head above the ear. The lower lip was considerably swollen, more, however, on the right than on the left side of the median line, and on its inner or mucous surface of the same side there was an apthous ulcer. She did not suffer any pain in the parts supplied by the ophthalmic and superior maxillary branches of the fifth nerve. The pain was paroxysmal, lasting from ten to fifteen minutes, and occurring after intervals of five or ten minutes, thus presenting the characteristic symptoms of tic douloureux in its worst form. She could not masticate any solid food, and even the act of swallowing or speaking would often induce a paroxysm. She got no rest by day and but little sleep at night, even with the aid of powerful anodynes. She had been admitted to the hospital on the fifth of September, fifteen days before I saw her, having dysentery, of which she was now cured. She did not come to hospital to get treatment for neuralgia, for she long before had deemed that incurable.

*Case* 1.—Her name was Margaret B., æt. 34 years, by occupation a housekeeper; married; and was born in New York. She dates her sufferings back fifteen years, when she was nineteen years old. Then, at the beginning of winter, she was suddenly and without apparent cause attacked with pains of a most excruciating character, which commenced in the lower lip, involved the whole course of the lower jaw of the right side, internal ear, and right side of head including right half of forehead.

No other part supplied with nerves by the ophthalmic branch of fifth pair, appeared to be the seat of pain, nor was any part supplied with the superior maxillary nerve involved. These pains were intermittent and recurred at intervals of fifteen or twenty minutes. Their intensity continued unabated for the three subsequent months, when they began to decline and gradually disappeared. The patient accounts for this departure of her neuralgia, by the occurrence of pregnancy, for in all her pregnancies, nine in number, she has observed, that, between the first and second month, the facial pains have begun to subside, and by the eighth month have entirely disappeared. Then, she always has remained free from neuralgia till from three to six weeks after confinement, when the pains have returned with all their original intensity.

In this way she suffered on through a period of about ten years, trying a great variety of remedies but without any benefit. At the end of this time, she succeeded in obtaining relief while under the care of a medical gentleman in New Orleans. The molar teeth (all sound) of the lower jaw of the affected side were extracted, but without any benefit. After that the supra orbital nerves were excised, together with branches of the portio dura going to the cheek from the parotid gland. The wounds were not allowed to heal for two or three weeks. Immediately after the operation she was salivated and then kept under the influence of mercury for four or five weeks. The pains continued with unabated severity for three weeks, when they began to subside, and at the end of two months entirely disappeared. This remission lasted two entire years. Thus relief was first brought to her by the operative surgeon, after ten years of useless trial of other means.

She was again attacked with the tic, about three years ago, while on a sea-voyage from New Orleans to New York, about three weeks after the birth of a still-born child. A great variety of remedies were again administered by different physicians, but without benefit, and she confessed to the taking of various patent nostrums recommended for nervous troubles. Failing to obtain relief from any source, she despaired of cure at the time of admission to the Hospital.

After careful examination it seemed to me clearly, that the nerve involved in the neuralgia was the inferior maxillary branch of the fifth pair, and that the seat of irritation producing the mischief was somewhere in the inferior dental branch, either in the filaments distributed to the lower lip, or in the canal of the jaw. I was led to this conclusion by the fact that *the pains always commenced in the lower lip and darted backwards along the course of the inferior dental nerve*, and then radiated over the side of the head. It occurred to me that an operation would be likely to benefit the patient, and that the removal of a portion of the inferior dental nerve from the canal of the lower jaw, afforded the best chance of success. I gave this opinion, in which Dr. Elliot coincided and he kindly transferred the patient to the division under my charge for surgical treatment. My colleagues unanimously sanctioned the proposition, and accordingly, on the 9th of October, 1854, the patient being under chloroform, I proceeded to operate, assisted by my friends Drs. Cane and Elliot, and in the presence of the House-Staff and other medical gentlemen.

An incision through the integuments was commenced about three quarters of an inch in front of the lobe of the right ear, and carried parallel to the ramus down to the base of the jaw a little in front of the angle, then continued forward along the base till the facial artery and vein were crossed, then carried upward parallel to the first incision, terminating below the track of Steno's duct. The facial artery and vein were exposed, tied, and then divided so as to save effusion of blood. The anterior portion of the parotid gland was turned back, the original incision continued inward through the masseter muscle, and the anterior portion ef the insertion of that muscle separated from the jaw. The tongue of flesh, thus formed with the knife, was raised, exposing the jaw over a considerable part of the inferior dental canal. The cavity of the mouth was not opened. A trephine of small size was then applied to the level surface of the jaw laid bare by the previous detachment of the masseter muscle, a button of bone removed, and the interior of the jaw exposed. The trephine was again applied a little in front of this spot and another button of bone taken out. In this way the inferior dental nerve was laid bare to the extent of fully one inch. It was then removed, and the spongy interior structure of the jaw scooped out along with it. This last step was executed for the purpose of obliterating completely that portion of the inferior dental canal, and for the further reason that a new bony formation would be likely to prevent a reunion of the excised nerve. A probe was then thrust into the dental canal and pushed as far as the mental foramen with a view to crush and destroy, as completely as possible, what remained of the nerve. The operation was now complete. The bleeding was easily stanched, the soft parts were brought into a proper position and secured by three points of interrupted suture and narrow strips of adhesive plaster. Cloths wet with cold water were ordered to be applied, and morphine in full doses internally. The patient suffered so much from the effects of chloroform that she did not fully recover her senses for two days. Her sufferings did not abate much for some time after the operation, although the pains were essentially altered in character. She could not open her mouth or laugh or smile, or move the muscles of that side of the face in any way without disturbing the wound, and suffering much from the soreness thereof. The pains continued very severe for a fortnight, and then gradually abated. In four weeks she was able to partake of solid food, (which she had not done for over a year) and was entirely free from pain in the face and head. She had become so much emaciated and weakened by the severity and long duration of her sufferings, that some time was required to completely re-establish her health. The only untoward occurrence during convalescence, was an attack of facial eryispelas, which was readily managed by the local use of lead and opium wash, and the internal administration of the infusion of rhubarb and soda, together with tonics. On leaving the hospital, the patient was particularly requested to report to us immediately if her neuralgia should return, as we were anxious to be informed with regard to the result of the case. About six months after the operation she came to the hospital of her own accord, to report that she continued free from suffering. And one year after the operation I met her in a railroad car when she appeared to be in perfect health, since which time I have neither seen nor heard from her. The presumption is that she is still well, although about two years have elapsed since the operation.

*Case 2.—Tic Douloureux of eight years standing—Removal of a portion of the superior maxillary nerve from the infra orbital canal, etc.*—

Martin M., æt. 29 years, born in Ireland, by occupation a carpenter and joiner, was admitted to Bellevue Hospital on the 19th day of September, 1856, for a very severe tic douloureux. He informed us that his family were not predisposed to any disease, and that no member of it had had neuralgia. He was attacked very suddenly about eight years ago, directly after shaving off his whiskers, and he attributes his difficulty to "catching cold in his face." He had intense lancinating pains, commencing generally in the right side of the nose or thereabouts, and shooting back along the track of the superior maxillary branch of the fifth nerve. Occasionally the pain began in right forehead. These pains occurred in paroxysms which lasted five or ten minutes, and recurred at short intervals. They were excruciating, and almost insufferable in character. Up to this time, his health had been good, and his habits strictly temperate. All the usual and many of the unusual remedies for tic douloureux, were administered to the patient by different physicians, but without any benefit. About five years ago, or in other words, about three years after his attack, a long incision through the scalp above the right ear was made by a distinguished western surgeon, but without affording any relief whatever. All the upper teeth on the right side have been extracted, but with the same result. About one year ago, (seven years after attack), he placed himself under my care. He was suffering very severely, having paroxysms every ten or fifteen minutes, which lasted five minutes. The paroxysms generally commenced with acute pains, beginning at a spot about midway between the right side of the nose and the infra-orbital foramen, and shooting back along the infra-orbital canal in the track of the superior maxillary nerve. The spot above mentioned, was tender and somewhat swollen. Touching it, immediately induced a paroxysm. Occasionally a paroxysm commenced with pain in the frontal branches of the supra-orbital nerve.

All other means having failed to afford benefit, and encouraged by the esult in Mr. B.'s case, I resolved to attempt an operation for this man's elief. I cut down upon the branch of the supra orbital nerve near the upra orbital notch, and excised the principal of them to the extent of bout three eighths of an inch. I then cut down upon the infra-orbital laments near to the infra-orbital foramen, with a view to treat them in similar manner, but the bleeding was so profuse and protracted, that I 'as unable to see them, and therefore had to content myself with simly dividing them. In a week after the operation, he was entirely free 'om the neuralgia, for the first time since his attack, which occurred bout seven years before. At this time I lost sight of him, and did not e him again till abont two weeks ago, when he called at my office. He ien informed me that he continued relieved for six months after the op-ration, and that the neuralgia returned shortly after the incision in his ieek had healed, or in other words, as soon as the divided nerves had nited. The paroxysms were very severe, appearing at intervals of ten r fifteen minutes, and lasting about five minutes. The pains seemed to egin in the spot already alluded to, about midway between the side of ie nose and the infra-orbital foramen, and to shoot back along the course the superior maxillary nerve to the Casserian ganglion, thence they diated, as from a centre, to the right side of the head, lower jaw, etc. 'hile reflecting on his case, and the great relief which the simple division the infra-orbital branches had afforded, it occurred to me that if I

could remove a portion of the trunk of the superior maxillary nerve from the infra-orbital canal, the relief produced by the operation would probably be of longer duration and might even be permanent. The patient stated his willingness to submit to almost anything which would be likely to rid him of his sufferings, and by my advice entered Bellevue Hospital, on the day already mentioned, for the purpose of having the operation performed.

The infra-orbital canal can be opened in two ways, so as to expose the superior maxillary nerve. The first method requires for its performance, the upper lip to be cut through, the cheek to be divided and dissected off from the upper jaw sufficiently for the antrum to be opened with a trephine. Then the roof of the antrum can be cut away in such manner as to expose the nerve. But it will be observed that this procedure requires the operation to be complicated by opening two cavities, viz.: the cavity of the mouth and the cavity of the antrum.

I practiced the other method in the following manner; my colleagues, Drs. Crane and Sayre, giving me efficient assistance. The patient being fully etherized, I commenced a semi-eliptical incision by entering the knife on a level with, and about three quarters of an inch external to, the outer canthus of the right eye, cutting through the skin and adipose cellular tissue, in a curved direction, downwards and inwards, almost to a level with the alæ of the nose, then cutting upwards and inwards, also in a curved direction, along the side of the nose, terminating the incision on a level with, and half an inch internal to, the inner canthus of the same eye. The incision was carried down to the malar and superior maxillary bones, and the oval flap carefully dissected up to the inferior margin of the orbit, thus freely exposing the spot where the nerve escapes from the infra-orbital foramen. The spouting vessels were tied as fast as they were exposed; I then proceeded to carefully detach with the handle of a scalpel, the flap from the margin of the orbit for a little distance backward, the eye-ball being at the same time carefully pushed upward by the fingers of an assistant, so as to give more room for the necessary manipulations. Then the flap and eye being carefully held up by two bent spatulæ; I applied a small sized trephine to the margin of the orbit, directly above the infra-orbital foramen, and removed a button of bone, thereby converting that foramen into a notch, and opening the anterior portion of the infra-orbital canal, thus exposing the trunk of the superior maxillary nerve. The trephine should be used with caution, for if too much force be employed, the operator will unwittingly push the instrument downward and backward into the antrum. A tenotomy knife was now thrust into the canal, and the nerve divided as far back as possible. The distal portion of the divided nerve was then turned forward and carefully removed. By this method, we excised fully a half inch of the nerve in question. We had troublesome hæmorrhage from the infra-orbital artery, which required the actual cautery to arrest it. A sharpened stick of lunar caustic was then thrust into the canal and held there for a couple of minutes for the purpose of destroying the nerve to a still greater extent. It will be observed that neither the cavity of the mouth nor the antrum was opened. The excised nerve appeared to be healthy at the point of division, but its external or distal portion was thickened, indurated, and reddened. The flap was replaced and secured by two points of interrupted suture, and the cold water dressing ordered. The patient was put upon a low diet, and directed to take morphine *pro re nata*. During the first day after the operation he had comparatively little pain, but on the second and third days his sufferings were very severe, which was probably due to the thorough cauterization of the nerve. On the fourth and fifth days the pains gradually abated, and on the sixth day he went out of the hospital relieved. Fully two-thirds of the wound had united by the first intention; the most depending portion was left to heal by granulation. The operation was followed by little or no constitutional disturbance. The eye was not interfered with, and the deformity was slight.

The patient promised to visit me, from time to time, in order to afford an opportunity to observe the progress of his case.—*New York Journal of Medicine.*

October 1, 1856.

